# Leveraging Sensor Technology to Characterize the Postural Control Spectrum

**DOI:** 10.3390/s24237420

**Published:** 2024-11-21

**Authors:** Christopher Aliperti, Josiah Steckenrider, Darius Sattari, James Peterson, Caspian Bell, Rebecca Zifchock

**Affiliations:** Department of Civil and Mechanical Engineering, United States Military Academy, West Point, NY 10996, USA

**Keywords:** postural control, postural stability, postural agility, dynamic stability, instability, force plate, IMU, accelerometer

## Abstract

The purpose of this paper is to describe ongoing research on appropriate instrumentation and analysis techniques to characterize postural stability, postural agility, and dynamic stability, which collectively comprise the postural control spectrum. This study had a specific focus on using emerging sensors to develop protocols suitable for use outside laboratory or clinical settings. First, we examined the optimal number and placement of wearable accelerometers for assessing postural stability. Next, we proposed metrics and protocols for assessing postural agility with the use of a custom force plate-controlled video game. Finally, we proposed a method to quantify dynamic stability during walking tasks using novel frequency-domain metrics extracted from acceleration data obtained with a single body-worn IMU. In each of the three studies, a surrogate for instability was introduced, and the sensors and metrics discussed in this paper show promise for differentiating these trials from stable condition trials. Next steps for this work include expanding the tested population size and refining the methods to even more reliably and unobtrusively characterize postural control status in a variety of scenarios.

## 1. Introduction

Human postural control (PC) is commonly understood to be the ability of an individual to maintain their center of mass (COM) within the limits of stability for a particular body pose or task [1]. PC is sustained by both the musculoskeletal and neurological systems of the human body, usually subconsciously, during both static and dynamic activities. Assessments of postural control are commonly employed to aide in the diagnosis of a wide range of dysfunctions in both systems. For example, mild traumatic brain injury (mTBI) lacks a definitive biomarker for diagnosis; however, it has been demonstrated that there is a correlation between impaired postural control and mTBI [2,3,4,5,6]. PC measurement has additionally been used in studies of muscle fatigue [7], spinal cord injury [8], lower limb amputee rehabilitation [9], and musculoskeletal instability [10]. Expanding the measurement modalities available for these evaluations will increase the utility of postural control to track and treat neurological and musculoskeletal impairments.

### 1.1. Postural Control Spectrum

**Figure 1:**
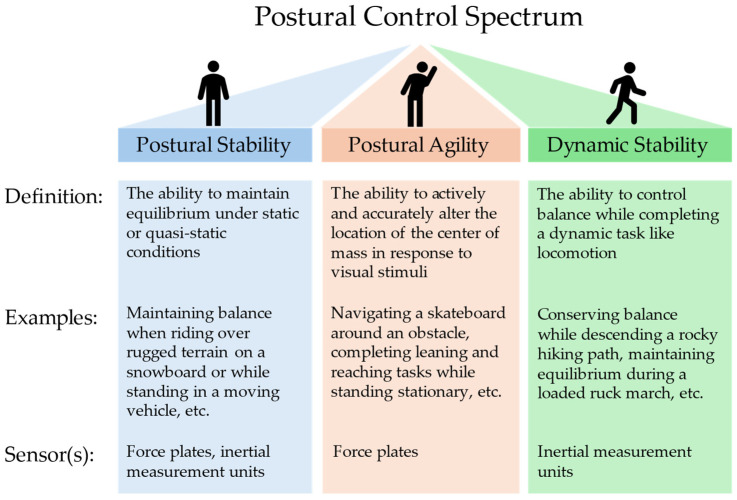
The three components of postural control as defined in this paper, examples of each element, and the sensors used to collect data for each in the presented study.

The overwhelming majority of research in the field of postural control deals with the ability of a person to maintain equilibrium under static conditions or quasi-static conditions (i.e., those with small localized perturbances); this class of PC research is commonly referred to as postural steadiness or stability (PS) [11]. Two less-studied aspects of postural control deal with the ability of a person to maintain balance when (1) actively and cognitively shifting their center of mass, and (2) dynamically propelling their body during tasks like locomotion or other more complex activities. In this paper, we defined these PC subdomains as postural agility (PA) and dynamic stability (DS), respectively, and we investigated the sensors and analysis techniques for evaluating and relating PS, PA, and DS. Figure 1 breaks down the distinction between the three aspects that comprise the postural control spectrum.

Maintaining balance for postural stability relies on proper sensory input from the cognitive, motor, and sensory neural networks [12]. Postural stability is evaluated during static tasks where the subject is either instructed to maintain a given position or recover to a given position following an external perturbation. While much of the current literature on postural stability has focused on center of pressure (COP) measurements derived from a force plate, there is a growing interest in the utility of accelerometers to measure a proxy for COM and its correlation to COP [13,14,15,16,17].

Postural agility is operationally defined in this paper as an individual’s capacity to actively and accurately alter the location of their center of mass in response to visual stimuli. This is derived from the definition of agility: “the ability to rapidly change body direction, accelerate, or decelerate” [18]. Postural agility is a unique aspect of postural control in that it involves conscious and deliberate adjustment of one’s center of mass to a new equilibrium. Postural agility is commonly employed in daily tasks such as reaching for objects or adjusting the body’s pose to avoid contact with obstacles in close quarters.

Dynamic stability is the term used in this paper to quantify balance control while completing a task that involves large-scale dynamic activity, especially locomotive tasks such as running or walking, which are predominantly cyclical in nature. Previous analyses of dynamic movements using wearable sensors have primarily been limited to movement recognition and classification techniques. Dehzangi et al. [19] leveraged convolutional neural networks powered by multi-sensor fusion to classify differing movement patterns. While dynamic stability is not directly related to movement type, a similar strategy could be employed to use accelerometer data to classify stability levels. Like the work by Dehzangi et al., other studies have extracted parameters from accelerometer data to characterize the stability of structures from their vibrational signals [20]. Additionally, measures of gait cycle frequency [19] and fast Fourier transform coefficients [19,21] have been used to characterize the variation and randomness of accelerometry data. However, previous work has been limited to use for the classification of stability during a variety of tasks, and the applications of these parameters for the acute identification of dynamic stability are limited. Furthermore, the optimized placement of a sensor on the human body, whose segments move cyclically with respect to each other, has not been explored. Due to its innately cyclical nature, gait may lend itself to the application of frequency domain parameters derived from accelerometry for the acute identification of dynamic stability.

The postural control spectrum can be holistically illustrated through the example of a skateboarder. Maintaining balance with both feet on the board while standing still or moving in a straight line is a test of the rider’s postural stability. Dynamic stability is required during kicking and pushing, as the rider needs to maintain balance while propelling their body forward. Finally, a rider needs to employ postural agility when executing a turn around an obstacle, which is accomplished by consciously shifting their center of mass away from equilibrium to cause the board to turn.

### 1.2. Sensors for Measuring Postural Control

While the characterization of postural control can be helpful for detecting and monitoring physical and cognitive impairment [22], the current methods are typically constrained to laboratory or clinical settings. This is due, in part, to the reliance on lab-calibrated force plates as the gold standard for measuring the COP as well as the limited use of postural agility and dynamic stability to assess postural control. Constraining measurements to these settings limits the ability of a researcher or clinician to detect emerging impairments that can impact an individual while they are executing an activity where impairment can occur such as while playing a sport or during a military engagement. Furthermore, limiting the characterization of postural control in these controlled settings reduces the generalizability of impairment detection to the actual environment where the individual must perform.

Methods of tracking and characterizing postural control in real-time, operational environments could improve the utility of this measurement. While wearable devices are used extensively for monitoring real-time biometrics, there is less focus on posture-derived metrics. Monitoring postural control can provide insights into the physical exertion levels, possible brain injuries, or vestibular conditions. This should include the appropriate instrumentation, analysis techniques, and environments to assess and characterize a given impairment.

The gold standard device for measuring data that can be used to assess postural control (especially postural stability) is the force plate. Most often, they are installed in the ground, and a subject will conduct a static or dynamic activity on them. While raw time-series force and moment data can be extracted from these devices, the most widely used metric delivered by force plates is the COP. The COP is the point where the net ground reaction force is applied [23] and represents how the neuromuscular system controls forces at the base of support. Although COP is not a direct measure of COM, the dynamic interplay between these two measurements is critical to understanding postural control, and the correlation between these values has been extensively explored [14,15,16]. In this study, we used the previously established measure of body sway as measured by accelerometers as a proxy of COM accelerations.

Another popular sensor for postural control assessment is the inertial measurement unit (IMU), which typically contains a three-axis accelerometer. These sensors have seen a tremendous increase in use for many biomechanical applications in recent decades as they can be small, wireless, and unobtrusively worn on the person. Compared to force plates, accelerometers offer a lightweight, wearable method for assessing postural stability in a variety of environments. The embedded accelerometers in smartphones have shown promise in detecting balance deficits in individuals with chronic strokes [24]. Current research suggests that sufficient features can be extracted from accelerometry data for quantifying postural control tasks [15]. However, most studies have used a single accelerometer, placed near the center of mass of the body, to measure body sway. It is possible that different locations of sensor placement on the body as well as data from multiple body-mounted sensors could improve the accuracy of these calculations.

Analyzed for future use in follow-on studies, surface electromyography (sEMG) and electroencephalography (EEG) are used to measure the complex muscular and neural mechanisms of postural control. sEMG is a non-invasive tool used to measure the activity of specific muscles. EEG is similarly a non-invasive sensor applied to a subject’s scalp that measures the electrical activity of the cerebral cortex during a task. These methods provide insights into the physiological activity contributing to postural control [9,25,26,27,28,29,30,31].

Video games have seen increasing use in the biomechanics and physical therapy communities over the last couple of decades, both for rehabilitative and evaluation purposes. By far the bulk of published research on incorporating video games with postural control research has been therapeutic in nature. Studies have found that devices like the Nintendo Wii Balance Board [32], Microsoft Kinect [33], other commercially available platforms [34,35], and research-grade immersive virtual reality systems [36] have produced improvements in postural balance and enhanced postural maintenance in elderly, disabled, and otherwise impaired individuals [37]. However, using video games as a tool to assess underlying posture-affecting pathologies is less common. Primary uses in the field thus far include evaluating concussions in unstable environments [38] and evaluating postural control strategies in healthy individuals [39]. The gamification of assessments chiefly allows for the measurement of cognitive involvement in postural control tasks to an extent that other research assessments do not.

In the sections below, we present our approach to characterizing the postural control spectrum by assessing each of the three PC components described above (PS, PA, and DS) using wearable and portable sensors. Our original contributions are as follows. First, we examine the number and placement of wearable accelerometers for assessing postural stability. Next, we propose metrics and protocols for assessing postural agility with the use of a custom force plate-controlled video game. Finally, we propose a method to quantify dynamic stability during walking tasks using novel frequency-domain metrics extracted from acceleration data obtained with a single body-worn IMU.

## 2. Materials and Methods

In order to assess the three elements of postural control, specifically the sensors and metrics used to quantify them, a pilot study involving a modest population of 11 participants was conducted. All participants were healthy, in their 20s, and gave their informed consent for inclusion before they took part in the study. The study was conducted in accordance with the Declaration of Helsinki, and with the research protocol that was reviewed by the United States Military Academy, Human Research Protections Program, CA-2024-102. The protocol was determined to be IRB-exempt.

### 2.1. Postural Stability

**Figure 2:**
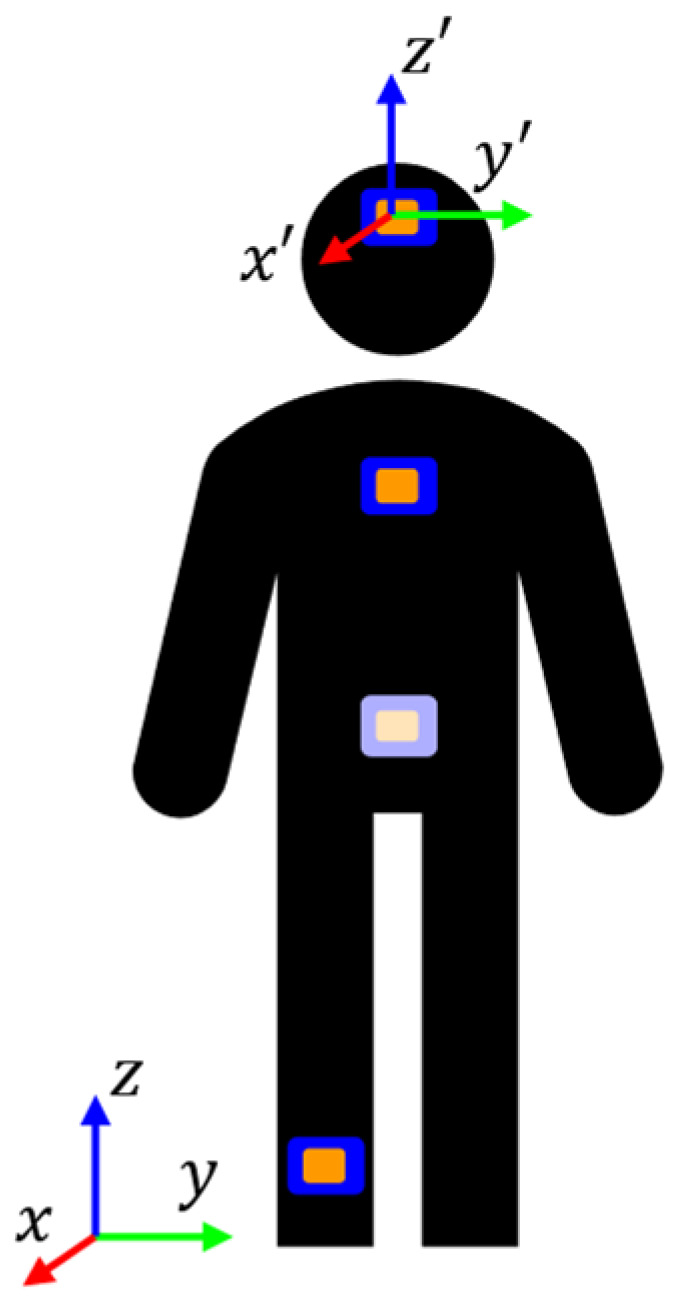
IMU placement. Lower-back IMU has been lightened to indicate placement on back of subject.

**Figure 3:**
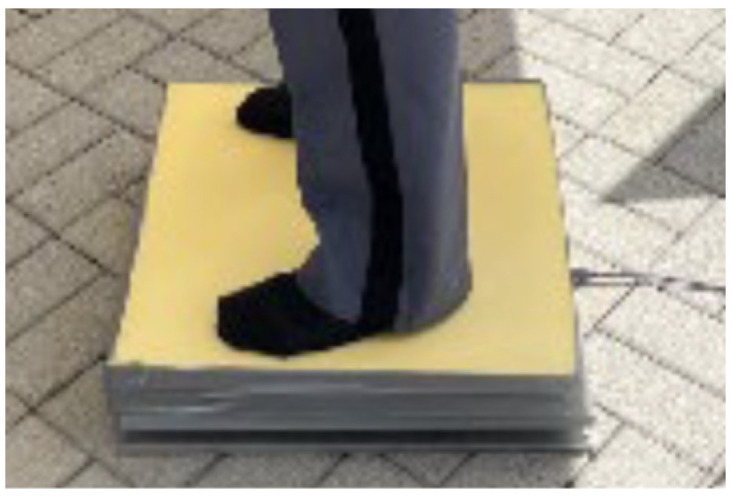
For some trials, the participants stood on a thick foam mat to elicit a destabilizing effect.

The purpose of the first part of the study was to assess the number and location of wearable accelerometers needed to maximize the accuracy of COP estimation. Data from six of the total participants were included, as complete datasets were unavailable from some participants. IMUs (I Measure U, Vicon; Denver, CO, USA) were placed on each participant’s forehead, sternum, lower back, and right ankle (Figure 2). These locations were chosen to represent each major segment of the body to understand how the linkages could move with respect to each other, even during stable tasks. The ankle sensor was only used to synchronize the accelerometer and force plate data in post-processing. Participants were asked to stand in their socks on an AccuGait-Optimized portable force plate (AMTI; Watertown, MA, USA) and place their hands at their sides while looking straight ahead at a visual target placed at eye level in front of them. They completed six trials in randomized order: feet apart on a hard surface, feet apart on a soft surface, feet together on a hard surface, feet together on a soft surface, standing single-legged on a hard surface, and standing single-legged on a soft surface. The soft surface was 7 cm-thick polyethylene foam with density of 18 kg/m^3^; this was intended to create a mild destabilizing effect (Figure 3). The inclusion of this range of destabilizing trials was included to elicit a variety of COP results for statistical analysis.

Force and moment data were collected from the force plate at 1000 Hz, and acceleration data were simultaneously collected from the IMU at 1125 Hz for 30 s. A quick hop at the start of each trial was used to synchronize the data from each sensor. Raw data were filtered with a fourth-order zero-phase Butterworth low-pass digital filter with a cutoff frequency of 10 Hz. The filtered force plate data were used to calculate separate COP measurements in the medial–lateral (ML) and anterior–posterior (AP) directions according to standard convention [40]. The body sway, a proxy for acceleration of the center of mass, from the head, lower back, and sternum accelerations were calculated using the method described by Ojie and Saatchi [17]. To summarize, the ML and AP coordinates can be simplified to:

(1) $$P=-\frac{a_{x}}{a_{z}}d_{z},\;ML=-\frac{a_{y}}{a_{z}}d_{z},$$

where $d_{z}$ is the height of the sensor from the ground and $a_{x,y,z}$ are the corresponding x/y/z accelerations measured at that sensor. The data from the sensors were each used to derive the total excursion, mean velocity, and the 95% confidence circular area of best fit, as previously described by Prieto et al. [41]. The total excursion and mean velocity were calculated as follows:

(2) $$TOTEX=\sum_{n=1}^{N-1}\sqrt{{\left({AP}_{n+1}-{AP}_{n}\right)}^{2}+{\left({ML}_{n+1}-{ML}_{n}\right)}^{2}},$$

(3) MVELO=TOTEX/T, 

where ${AP}_{n}$ and ${ML}_{n}$ are the $n^{th}$ data points of the AP and ML coordinates, N is the size of the time-series data, and T is the total time of the trial. The calculation for $AREA_{cc}$, the 95% confidence circle, which is more involved, can be found in the work by Prieto et al.

**Figure 4:**
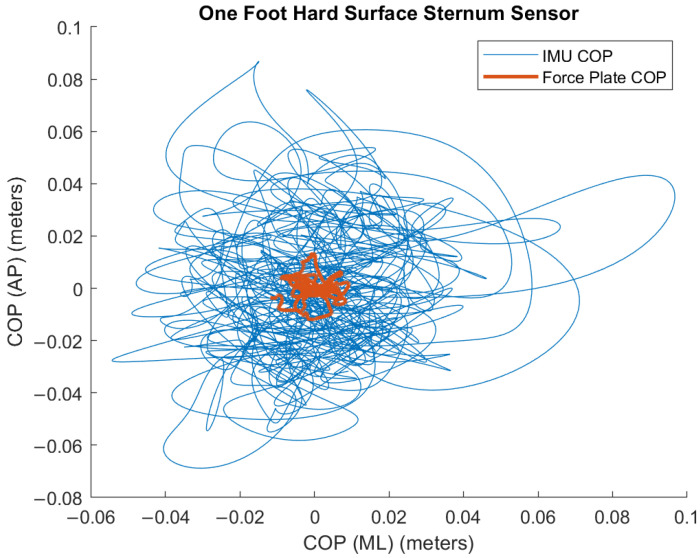
IMU (accelerometer)-derived trace (blue) with force plate-derived COP trace (red) for an example trial. Note the different range and shapes of the traces.

Since the force plate and the IMUs’ accelerometers have “different sensing principles, bandwidths, and geometric references”, the body sway, as traced by the IMUs, and the COP calculated from the force plate are incongruent [15]; Figure 4 confirms this notion. For this reason, correlations were examined to identify relative, as opposed to absolute, agreement between the metrics derived from the different sensors.

### 2.2. Postural Agility

**Figure 5:**
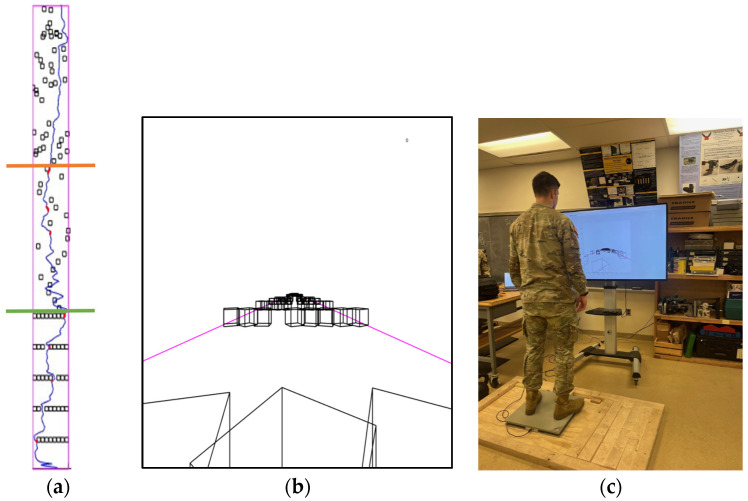
(**a**) Aerial view of the postural agility assessment game. The green line denotes the transition from stage 1 to stage 2 and the orange line denotes the transition from stage 2 to stage 3. The blue line depicts the path being traced by the subject’s center of pressure on a force plate. The red points are where the subject failed to avoid an obstacle and incurred a penalty (note that these may not directly coincide with the blocks depicted in stage 2 at this instant because the blocks are moving). (**b**) Image of gameplay. The magenta lines are the boundaries of the course, and the blocks (rotating clockwise) are the obstacles that the subject is instructed to avoid. A counter in the top right of the screen increases as penalties are incurred to provide feedback to the subject. (**c**) Test setup consisting of a portable (USB-powered) force plate and screen.

The purpose of the second part of the study was to propose a method to evaluate postural agility using a real-time interactive assessment. This assessment consisted of a custom force plate-controlled video game written in MATLAB (Mathworks; Natick, MA, USA). When introduced to the game, the subject was outfitted with IMUs as described in Section 2.1 and instructed to stand on the force plate (Figure 5c). The subjects then actively shifted their center of mass side-to-side to avoid obstacles that appeared to be moving toward them on a large screen. Figure 5b provides a screen capture of the first-person-view game, where obstacles are rotating black wireframe boxes in a white world with magenta lines bounding the edges of a path. The blocks move down the path at a constant rate while the virtual medial–lateral movement of the player is controlled by the force plate COP (accelerometry data were also collected but not currently used). When the player goes beyond the bounds of the arena or collides with a block, the boxes turn red to indicate a penalty to the subject.

The game consists of three stages, each designed to evaluate different metrics of postural agility. These stages are identified in the aerial view of Figure 5a. In the first stage, the blocks appear in regularly spaced rows across the path perpendicular to the subject, with a random gap in each line through which a subject can avoid colliding with a block. This stage allows the game to have some control over the subject’s movement, where the speed and accuracy with which the subject transitions from one gap to the next can be evaluated. Assessments of dynamic transition tasks such as this have importance in the literature for postural control evaluation [42]. The subject is expected to have a stable COP to get through the gap in one row before then shifting and stabilizing their COP in a single motion to a new position. The second stage consists of single blocks evenly spaced down the arena that slide back and forth across the stage perpendicular to the direction of travel. This part of the game assesses the subject’s short-horizon ability to respond to visual stimuli and quickly adjust their COP as well as their cognitive ability to predict what position the blocks will be in when they pass. The final stage consists of static blocks whose positions are randomly generated. This stage assesses the subject’s long-horizon path planning ability as well as their fine postural agility, as the passage between any adjacent blocks is not guaranteed to be large.

Naturally, as with any skill-based game, learning can be a significant component of performance. Thus, each subject completed the game three times standing directly on the hard force plate surface and three times on the same 7 cm-thick foam pad, as shown in Figure 3. This allowed the research team to not only elucidate any learning effects on the subject’s score, but also to obtain data from a proxy unstable condition. To avoid intentionally training on increasingly challenging versions of the game, a coin toss was used to determine whether the subject completed the hard-surface or soft-surface trial first.

Six metrics were extracted from each trial of the postural agility assessment, as summarized in Table 1. The first was a simple penalty that indicates the percentage of the game during which the subject collided with boxes or outside the bounds of the arena. This value depends on the postural agility of the subject but is also strongly affected by the game’s cognitive aspect, which the subsequent parameters do not consider to the same degree. The total excursion ($TOTEX_{ML}$) [41] and mean acceleration ($mACC_{ML}$) of the subject’s center of pressure were measured in the medial–lateral direction. Because the subjects were directed to control their center of mass in the medial–lateral direction, these metrics provide an insight into the subject’s ability to actively manipulate their posture away from equilibrium in response to external stimuli. The total excursion and mean velocity of the subject’s center of pressure in the anterior–posterior direction ($TOTEX_{AP}$ and $mVELO_{AP}$, respectively) were additionally recorded. Since the subject was given no instruction about their movement in this direction, these metrics measure the subject’s unconscious ability to maintain postural control in the orthogonal direction during deliberate side-to-side movement. Finally, the mean time to new stability point (mTNSP) [43] was measured during the first stage of the game to assess the subject’s ability to transition from one stable equilibrium posture to the next between gaps in rows of blocks.

### 2.3. Dynamic Stability

**Figure 6:**
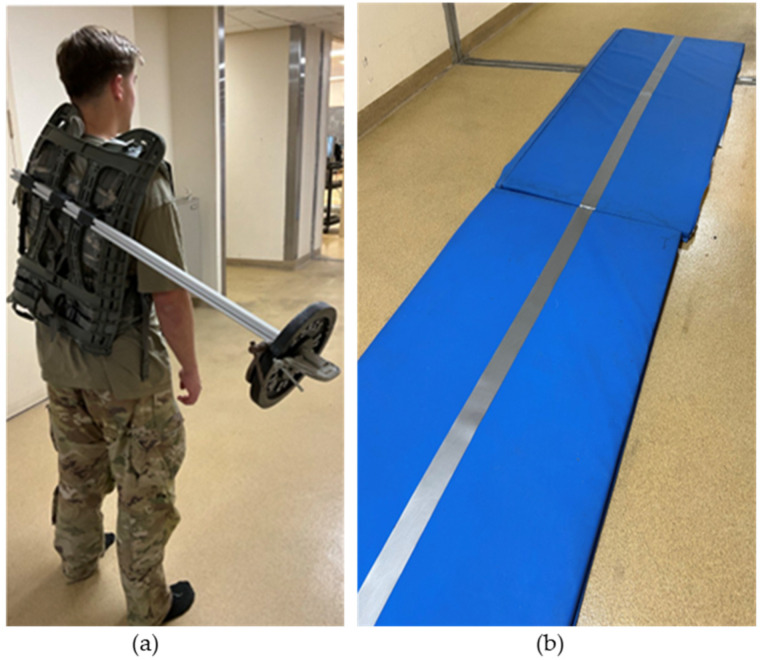
(**a**) Asymmetric loading condition for dynamic stability trials. (**b**) Foam walking surface with specified centerline for dynamic stability trials.

The purpose of the third part of the study was to propose a method to detect differences between stable and increasingly unstable dynamic walking tasks using a single body-worn accelerometer. IMUs were placed on each participant’s forehead, sternum, lower back, and right ankle, as discussed in Section 2.1 (Figure 2). Participants were asked to walk in their socks along a 15 m straight path in each of four randomized conditions. For two of the conditions, participants walked on a hard surface both unloaded and asymmetrically loaded using a 10 lb weight affixed to a backpack frame at a distance of 0.7 m from the body midline, with hip and chest straps secured (Figure 6a). The same two unloaded/asymmetrically loaded conditions were also executed along a 13 m soft mat that was 3.5 cm-thick with a tape line down the center, along which the participant placed their feet as they walked (Figure 6b). The distance was intentionally shorter to account for the slower pace and decreased step lengths on this surface. During each of the walking trials, accelerometer data were collected at 1125 Hz, and only data relative to the participant’s medial–lateral axis were used for further analysis.

**Figure 7:**
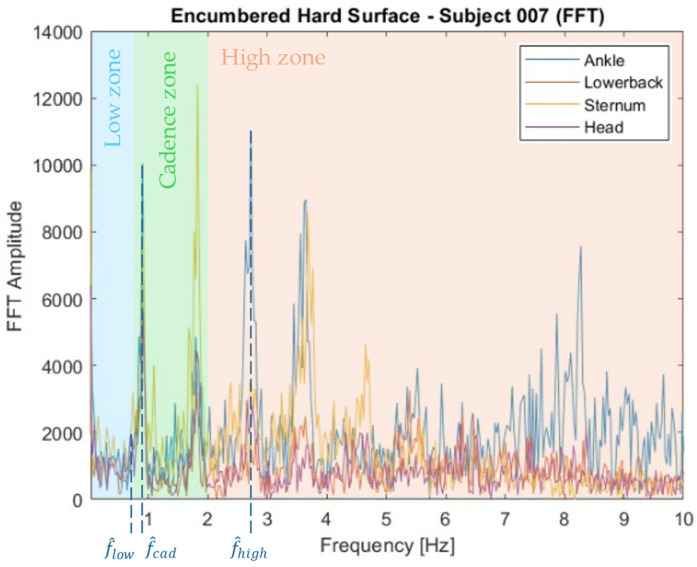
Acceleration FFT plots for four IMU placements, frequency zones, and peak frequencies for the ankle case (blue) of a single subject.

Raw accelerometry data were converted to the frequency domain using fast Fourier transform (FFT) after the DC offsets were first subtracted. To extract meaningful features from this frequency domain dataset, three frequency zones were established. The “low” walking frequency zone ranged from 0 to 0.8 Hz, the “cadence” zone ranged from 0.8 to 2 Hz, and the “high” zone ranged from 2 to 10 Hz. These zones were used based on the well-documented fact that the unencumbered human gait tends to occur near 1 Hz [44], while signals much higher than 10 Hz are unlikely to have a musculoskeletal origin. Within each zone, the frequencies corresponding to the highest amplitude peaks (${\hat{f}}_{low}$, ${\hat{f}}_{cad}$, and ${\hat{f}}_{high}$, respectively) were found. Figure 7 shows these peak frequencies for the ankle accelerations as an example case, though the peak frequencies in the three zones were extracted from each sensor location. The low-to-cadence and cadence-to-high frequency ratios $r_{l:c}$ and $r_{c:h}$ were then calculated as follows:

(4) $$r_{l:c}=\frac{{\hat{f}}_{low}}{{\hat{f}}_{cad}},\;r_{c:h}=\frac{{\hat{f}}_{cad}}{{\hat{f}}_{high}}.$$

It was hypothesized that these proposed nondimensional frequency-domain metrics could contain valuable information about the stability of a subject while walking, as non-cadence sway at low or high frequencies could indicate imbalance. Comparisons of each frequency ratio were made using a two-way, repeated-measures ANOVA to examine the effect of sensor location and surface condition.

## 3. Results

### 3.1. Postural Stability

**Figure 8:**
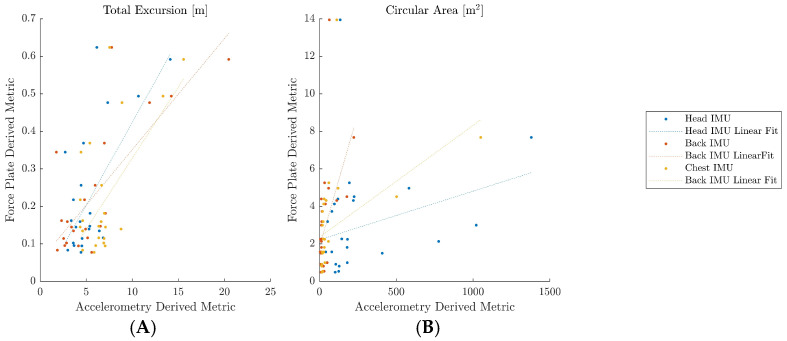
Correlation values for the head, back, and chest sensors for total excursion (**A**) and circular area of best fit (**B**).

The initial results of the postural stability investigation are shown in Figure 8. The magnitudes of the COP-derived variables derived from the force plate were similar to previously published values [41]. Mean velocity calculations were within a 13% difference of previous data, while the values for total excursion differed slightly because the data were analyzed for 15 s rather than 20 s. As seen in Table 2, the total excursion had a strong correlation for all three sensor locations. Of the six features, only the circular area from the head mounted IMU showed a poor correlation to the force plate-derived metrics. Of the three sensor locations, the accelerometer placed on the subject’s lower back provided the highest correlation with the force plate data. Additional research is ongoing to determine whether sensor data from multiple accelerometer locations can be combined to further improve the accuracy of the calculations.

### 3.2. Postural Agility

**Figure 9:**
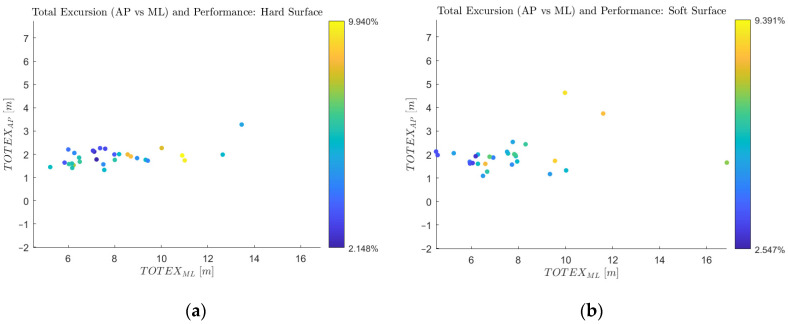
Medial–lateral and anterior–posterior total excursions for (**a**) hard and (**b**) soft surfaces.

Data from 10 of the 11 total participants were included in the results presented below. Figure 9 shows the total excursions of the 30 hard-surface and 30 soft-surface trials (three per subject). The color of each datum corresponds to the penalty incurred by the subject while playing the game (the lower the penalty, the higher the performance on the game). As expected, there was substantially greater excursion in the medial–lateral direction than the anterior–posterior direction on average. The range of every subject’s conscious side-to-side motion exceeded their subconscious forward–backward motion as they played the game. Furthermore, while much more data are necessary to fully populate the feature space, there is an apparent trend that a $TOTEX_{ML}$ of 6–7 m corresponds with optimal game performance. For the hard-surface condition, this apparent optimum was slightly larger than for the soft-surface. The worst-performing participants tended to have large $TOTEX_{ML}$ scores, and $TOTEX_{AP}$ appeared to have a larger correlation with poor performance when the surface was soft. Table 3 summarizes the averages of all metrics for hard and soft conditions.

The soft surface was introduced as a surrogate for instability. The table shows that, although minimal, there was an intuitive trend differentiating subject performance between the stable and unstable cases. The soft-surface penalty score was slightly larger, in addition to the mTNSP, $mACC_{ML}$, and $TOTEX_{AP}$. Conversely, the average $mVELO_{AP}$ and $TOTEX_{ML}$ were slightly smaller for the soft surface, which may be an artifact of the foam mat dampening the input to the force plate rather than an indication of instability.

**Figure 10:**
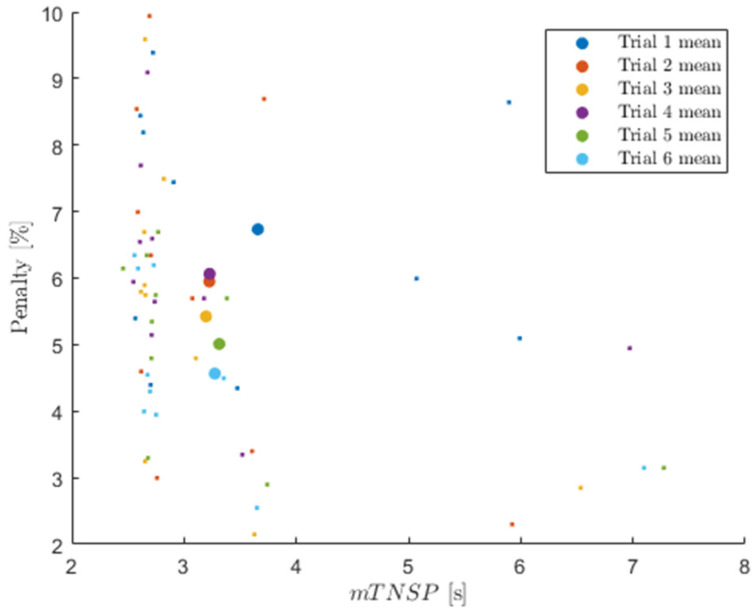
Penalty vs. mTNSP for all trials including means, with trial number coded by color.

Figure 10 presents the penalty score and mTNSP for all subjects and trials, with large dots indicating the mean over all subjects for each trial number. As the figure shows, the penalty scores consistently decreased (with the exception of trial 4), which demonstrates an overall improvement in performance. There was also a general reduction in mTNSP from trial 1 to trial 6, though this trend was much weaker. The penalty and mTNSP did not appear to show any strong correlation.

### 3.3. Dynamic Stability

**Figure 11:**
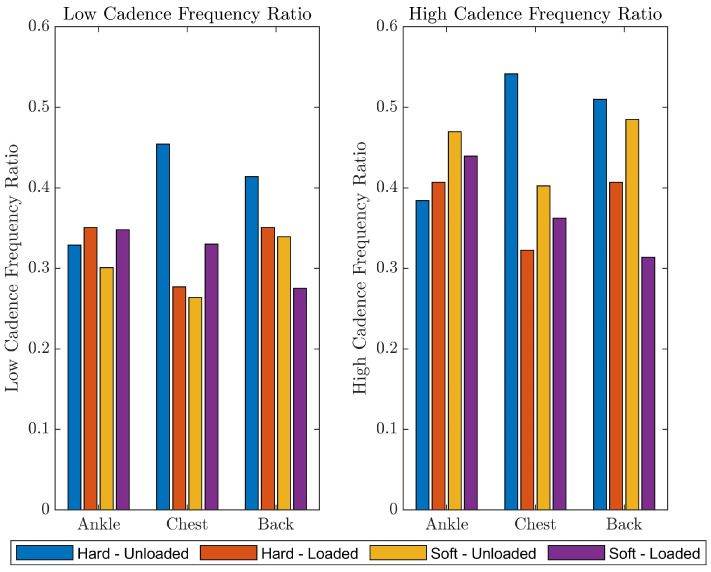
Frequency ratios for low-cadence and high-cadence, for the ankle, chest and head sensors in the four dynamic walking trials.

The initial results of the dynamic stability trials are shown in Figure 11. These findings suggest that $r_{l:c}$ and $r_{c:h}$ may be useful for detecting dynamic instability. Due to a systematic issue with the sensor on the lower back, insufficient data were available for that sensor location. The ANOVA suggested that there was a significant interaction between sensor location and surface for $r_{c:h}$ (*p* < 0.01), and a trend toward an interaction for $r_{l:c}$ (*p* = 0.12). This suggests that the sensors may have varying capacity to detect differences between walking surfaces and that the chest and head sensor locations may be particularly useful for detecting differences between relatively stable (hard surface, unloaded) and unstable (soft surface, asymmetrically loaded) conditions. For both frequency ratios, the hard surface, unloaded condition showed higher ratios than the soft surface, asymmetrically loaded condition, suggesting that the peak amplitudes were closer to the frequency of gait. The other two walking conditions tended to show ratio values that were between the most stable and unstable conditions, but there was not a clear trend.

Ongoing work on this topic will include data for the lower back sensor and will explore the use of Welch’s power spectral density estimate to characterize the frequency domain as well as the spectral centroid and spectral variance as extracted features [20].

## 4. Discussion

### 4.1. Postural Stability

The goal of this part of the study was to determine the best location on the human body to place accelerometers that would produce the most accurate correlations with the force plate COP calculations as well as the best metric for determining the correlation between the accelerometer and force plate data. The correlational statistics suggest that, of the metrics derived from accelerometry, the total excursions and mean velocity were the most predictive of the force plate metrics. Additionally, the sensor placement on the lower back appeared to exhibit the closest relationship with the entire body, as measured by the force plate data. This is not entirely surprising, as the lower back is close to the COM and is similar to where many previous studies have mounted single sensors [15,24,45]. However, it is surprising that the sensor placement on the chest demonstrated the lowest correlations. Given the fact that both the back and chest sensors were on a single body segment (the trunk), it was expected that they would have similar relationships with the force plate data, while the head would have more independent findings. Future research with additional participants is necessary to understand the strength of these relationships. Additionally, future research is needed to understand whether the inclusion of combined data from multiple sensors can significantly improve the prediction of postural control metrics. Improvements in the capacity of lightweight, inexpensive sensors to assess postural stability will allow for easier, more frequent assessment on athletic fields and in tactical environments, and can improve care for physical and cognitive impairment.

### 4.2. Postural Agility

There are several observations pertaining to the preliminary postural agility assessment findings that are worth noting. First, there was an apparent increase in the overall variation of TOTEX values as the standing surface softened, as evidenced by Figure 9. Especially obvious in the soft-surface condition, higher values of $TOTEX_{AP}$ may correspond with poorer performance on the game. This suggests that the game may help reveal instability in the body plane perpendicular to conscious movement. Figure 9 also demonstrates that subjects were largely able to decouple their medial–lateral and anterior–posterior movement in quasi-static activities such as the game used here.

The differences between the hard-surface and soft-surface values shown in Table 3 support a conjecture that the firmness of the ground, as a proxy for instability, is correlated with a player’s performance, albeit minimally. It is worth noting that the polyethylene foam used across this study was relatively firm, and it is likely that a softer and thicker destabilizing surface would have a stronger effect on stability and agility. Remarking on the small differences that are apparent in Table 3, there is again an agreement with intuition. Game performance could be reasonably predicted to suffer, confirmed by the increase in penalty score, and there was a ~0.1 s average delay in response time to stimuli as measured by mTNSP. A reduction in $TOTEX_{ML}$ likely corresponded to a difficulty in stretching the COP to the edges of the force plate to avoid blocks in the game when the foam surface was present, while the increase in average $TOTEX_{ML}$ can be reasonably assigned to destabilization in the passive body plane. Intuitive explanations for changes in $mVELO_{AP}$ and $mA{CC}_{ML}$ are more elusive, but these metrics may prove to provide insight or have predictive power in the future of this research.

### 4.3. Dynamic Stability

The results of this study present preliminary findings to suggest optimal sensor locations and frequency-based features that can be extracted to determine the dynamic stability levels. Metrics derived from accelerometry data in the frequency domain may be a useful tool for identifying deviations from stable movement patterns, particularly for cyclical tasks such as walking. While these findings are promising, this section of the study requires additional data, as the current results did not include data from the lower back sensor. The findings from the postural stability work in this study suggest that this location might be optimal for characterizing COP, which could translate to the characterization of dynamic stability using frequency-based metrics.

### 4.4. Emerging Sensors for Future Integration

Subsequent iterations of this protocol will include additional sensing modalities to capture the musculoskeletal and neural mechanisms of postural control more robustly. Using the existing protocols, surface electromyography (sEMG) recordings will be taken from the gastrocnemius medialis, tibialis anterior, rectus femoris, and soleus during postural control tasks [46,47]. These muscles demonstrate the most active recruitment during both active postural control and maintenance of postural stability. Electroencephalography (EEG) measurements from electrodes on a standard scalp EEG cap in the 10–20 internal standard configuration are sufficient to produce discernable cortical features relevant to postural control tasks [30]. Deriving correlations between the intended posture state (EEG), physiological actuation to achieve this intention (EMG), and the resulting postural state (force plate and IMU) is a critical step toward developing a complete understanding of the postural control spectrum, allowing for directed treatment and the future development of neural controlled assistive devices.

## 5. Conclusions

Accelerometers are already built into several body-worn devices for athletic and tactical endurance tasks. The use of these signals to understand postural stability can help improve the identification of instabilities that may be indicative of acute or chronic fatigue or injury. This study proposed a three-part protocol for assessing the spectrum of postural control using wearable and portable sensors. The first part leverages the well-established force plate method as a baseline to evaluate the effectiveness of accelerometry via IMU for the evaluation of postural stability to occur outside the lab environment and provides insights into how relative movements and the position of different body parts affect the global center of mass. While others have used accelerometry for similar postural assessments, this study specifically examined the effect of sensor placement on postural stability evaluation. Utilizing the same sensor configuration in the dynamic stability phase similarly allowed the study to be executed expeditiously. A final contribution of this work is the newly defined subset of postural control called postural agility. Classical postural control studies focus on maintaining a stable center of mass, either at rest or during a dynamic task, whereas this study assessed a subject’s ability to intentionally adjust their center of mass to establish a new stability away from equilibrium in one plane while subconsciously maintaining their stability in another. For all three stages, the introduction of destabilizing surfaces and stimuli provides both a baseline for how a subject will perform with impaired postural control, and insights into the mechanisms involved in re-establishing their center of mass during a dynamic task. The overlap in these protocols connects the three components of postural control that were outlined in this study and provides data to examine their relationship and how they combine to give a complete picture of a subject’s postural control.

### 5.1. Limitations

The intent of this paper was to describe a method of collecting and analyzing data to explore the spectrum of postural control. However, the presented results were based on a small sample size of healthy individuals with an imposed external stimulus to create instability. Thus, the generalizability of the findings are somewhat limited until more data can be obtained. While these initial results suggest that these methods may be useful for distinguishing between stability states, additional research with larger sample sizes is needed to clarify the utility of these methods to distinguish between various populations with different levels of either intrinsic or extrinsic instability.

It is also important to address the versatility of the requisite sensor suite of the proposed methods, or more specifically, when that versatility breaks down. The size and toughness of modern wearable accelerometers continue to improve as their usefulness in athletic and military contexts becomes more widely accepted. Today, wearable IMUs can withstand nearly anything the human body can. However, there are more restrictions on the environments in which force plates provide accurate information. The COP readings delivered by a force plate are only as reliable as the ground on which it rests being solid, flat, and level. Testing accommodations can often be made with additional peripheral equipment, but this can limit the versatility of the sensor. These limitations serve to highlight the preferability of wearable IMUs for PC assessment, and by extension, the importance of using accelerometer-derived metrics as surrogates for COP metrics.

### 5.2. Future Work

This protocol facilitated a wide range of data collection to understand the full spectrum of postural control in a single session lasting approximately 30 min. Subsequent participants may be instrumented with the additional sensors discussed in Section 4.4 to examine the intention and internal actions preceding the center of mass adjustments seen by the current sensors. However, each of these protocols was also designed to be used independently for the assessment of the specific postural control measure of interest as well as in environmental conditions of interest.

Future work will consist of further testing with the full suite of sensors on a larger body of participants in order to (1) discover correlations between sensing modalities, (2) understand neural and musculoskeletal mechanisms of postural control, and (3) establish criteria for using this protocol as an evaluation tool. A desirable end state would be a comprehensive suite of unobtrusive wearable and portable sensors that all provide data to an improved model that delivers insights into the postural stability, postural agility, and dynamic stability.

## Figures and Tables

**Table 1:** Postural agility metrics.

Metric	Source	Target	Units
Penalty	Game feedback All stages	postural agility, cognition	Percent of game
$\mathrm{Total}\;\mathrm{excursion}\;\mathrm{of}\;\mathrm{medial}–\mathrm{lateral}\;\mathrm{COP}\;(TOTEX_{ML}$)	Force plate All stages	active postural agility	Meters
$\mathrm{Mean}\;\mathrm{acceleration}\;\mathrm{of}\;\mathrm{medial}–\mathrm{lateral}\;\mathrm{COP}\;(mACC_{ML}$)	Force plate All stages	active postural agility	Meters per second per second
$\mathrm{Total}\;\mathrm{excursion}\;\mathrm{of}\;\mathrm{anterior}–\mathrm{posterior}\;\mathrm{COP}\;(TOTEX_{AP}$)	Force plate All stages	passive postural stability	Meters
$\mathrm{Mean}\;\mathrm{velocity}\;\mathrm{of}\;\mathrm{anterior}–\mathrm{posterior}\;\mathrm{COP}\;(mVELO_{AP}$)	Force plate All stages	passive postural stability	Meters per second
Mean time to new stability point (mTNSP)	Force plate Stage 1	recovery to stable position following rapid transition	Seconds

**Table 2:** Postural stability model parameters and correlation coefficients.

Feature	r^2^	m	b	Correlation Coefficient
Total Excursion—Head	0.4152	0.0441	−0.0166	0.6616
Total Excursion—Back	0.5985	0.0296	0.0551	0.7835
Total Excursion—Chest	0.3265	0.0378	−0.0504	0.5936
Circular Area—Head	0.0453	0.0025	2.2142	0.2888
Circular Area—Back	0.2433	0.02841	1.8275	0.6974
Circular Area—Chest	0.1608	0.0059	2.3554	0.5983

**Table 3:** Average metrics for hard and soft surface conditions.

	$TOTEX_{ML}$	$TOTEX_{AP}$	$mVELO_{AP}$	$mACC_{ML}$	mTNSP	Penalty
**Hard**	7.981 m	1.889 m	0.923 mm/s	12.227 mm/s^2^	3.260 s	5.541%
**Soft**	7.663 m	1.967 m	0.879 mm/s	15.455 mm/s^2^	3.372 s	5.708%

## Data Availability

The original contributions presented in the study are included in the article/Supplementary Materials; further inquiries can be directed to the corresponding authors.

## Supplementary Materials

The following supporting information can be downloaded at: https://www.mdpi.com/article/10.3390/s24237420/s1, code and data available upon request.
